# Early Experience with Intravitreal Bevacizumab Combined with Laser Treatment for Retinopathy of Prematurity

**DOI:** 10.4103/0974-9233.65500

**Published:** 2010

**Authors:** Ayesha E. Ahmed, Roomasa Channa, Jibran Durrani, Azam Ali, Khabir Ahmad

**Affiliations:** Department of Ophthalmology, Aga Khan University Medical College, Karachi, Pakistan; 1 Department of Surgery, Aga Khan University Hospital, Aga Khan University, Karachi, Pakistan

**Keywords:** Intravitreal Bevacizumab, Retinopathy of Prematurity, Laser treatment

## Abstract

**Objective:**

This study was designed to present our early experience with intravitreal bevacizumab combined with laser treatment for retinopathy of prematurity (ROP) at a single institution over a 13-month-period.

**Methods:**

A retrospective case series of eight children with ROP who received intravitreal bevacizumab combined with laser treatment between June 2007 and July 2008 were reported. A chart review was conducted to evaluate if stability of the ROP lesion had been achieved. Main information collected included data on demographics, gestational age, birth weight, length of stay in neonatal intensive care unit, and stage of ROP.

**Results:**

Fifteen eyes of eight subjects were treated. One eye did not receive any treatment due to complete retinal detachment. The median age at treatment was 8 weeks (range, 6 weeks to 1 year). The most common stage of ROP was 3+. All eyes remained stable at 1 year or later after treatment.

**Conclusion:**

Intravitreal bevacizumab in conjunction with laser treatment had promising results at our institution. We recommend prospective, randomized, controlled clinical trials to compare the effect of laser treatment alone, of bevacizumab treatment alone (at different doses), and of combined bevacizumab and laser treatment.

## INTRODUCTION

Retinopathy of prematurity (ROP) is a leading cause of childhood blindness worldwide. The first known description of the condition was in the 1940s, and an association with prematurity was identified.^1^ No treatment was available at that time. In recent years, cryotherapy and laser therapy have been used with limited success. Although these ablative treatments reduce the incidence of blindness by 25% in infants with severe disease, visual acuity post-treatment remains considerably impaired.^2^

The role of vascular endothelial growth factor (VEGF) in the pathogenesis of ROP has been identified. It is believed that exposure to high levels of oxygen during the neonatal period leads to obliteration of vessels. Vaso-obliteration leads to ischemia which further leads to the release of vascular growth factors and neovascularization.^3^ VEGF increases permeability of vessels^4^ and promotes endothelial cell proliferation.^5^ Transcription of VEGF mRNA increases with hypoxia and has been identified to play a vital role in retinal angiogenesis.^6^ Recent studies have identified that insulin-like growth factor 1 (IGF-1) plays a permissive role—in its absence VEGF is unlikely to fully stimulate vessel growth.^2^

Knowledge of the molecular mechanisms of the pathogenesis of ROP has become the basis for current therapeutic approaches. Angiogenic factors, inhibitors of basement membrane changes, endogenous inhibitors such as pigment epithelium-derived factor, and anti-inflammatory drugs are all potential avenues for treatment.^7^ Anti-VEGF has been successfully used in diseases such as neovascular AMD, proliferative diabetic retinopathy. Studies using bevacizumab for the treatment of ROP with encouraging results have recently been published.^8^–^10^ Bevacizumab is an anti-VEGF monoclonal antibody.

Intravitreal bevacizumab injections have recently gained popularity as a potential treatment for several intraocular neovascular diseases without known serious ocular systemic adverse events.^11^ The use of intravitreal bevacizumab for severe ROP combined with the use of indirect argon laser is the main focus of this study.

## MATERIALS AND METHODS

We retrospectively viewed the clinical records of 15 consecutive eyes of eight subjects suffering from ROP, treated with intravitreal bevacizumab injections combined with laser treatment. All cases underwent treatment from June 2007 to July 2008 by one surgeon.. All eyes had an increased risk of tractional retinal detachment(AA, Aga Khan University Hospital, Karachi). Inclusion criteria were 32 weeks of gestation or less and birth weight of 1300 g or less. All eyes had stage 3 to stage 4 ROP were at a high risk of permanent vision loss and had a decreased likelihood of improvement with conventional laser therapy alone. The ROP stage and plus disease were defined on the basis of the international classification scheme.^12^ Exclusion criteria were refusal of informed consent from a parent or guardian. This meant that an infant would receive laser therapy only. The peripheral neovascular activity was evaluated in each eye by indirect ophthalmoscopy. Changes in leakage, tortuosity, and dilatation of retinal vessels were evaluated by two different consultants (specialists *in vitro*-retina surgery), neither of whom performed the surgery(vitreo-retina specialist and a corneal specialist). Following indirect argon laser therapy, a dose of 0.625 mg of bevacizumab, i.e., half of the adult dosage, was injected intravitreously with the patients under general anesthesia in cases where the bevacizumab was injected after laser therapy [Table 1]. The follow-up period ranged from 3 to 8 months. Vitrectomy was not performed on any eye of the cohort.

**Table 1:** Preoperative characteristics and treatment of eight subjects with retinopathy of prematurity that underwent laser therapy and intravitreal bevacizumab injection

Case #	Sex	Date of birth	Zone	Stage of ROP	Birth weight (g)	Gestational age (weeks)	Postmenstrual age at the time of laser (weeks)	Postmenstrual age at the time of injections (weeks)	Eye
1	M	08.07.08	2	4a+,3+	860	27	33	33	OU
2	F	05.03.08	2	3+,4a	820	29	36	36	OU
3	F	29.08.07	3	3,3	1200	29	37	37	OU
4	F	13.04.07	1	3+,3+	1000	32	38	38	OD
5	M	22.11.07	2	3,3	1200	29	36	38	OU
6	F	20.06.07	2	3,3	800	29	38	38	OU
7	M	01.06.07	2	3,4b	1220	29	37,41	37,41	OU
8	M	20.04.07	2	3+,3+	1020	29	37	37	OU

OU - bilateral; OD - right eye; OS - left eye; M - male; F - female

## RESULTS

One eye did not receive any treatment of a complete retinal detachment. The patient characteristics are shown in Table 1. The study included four females and four males [Table 1]. The gestational ages ranged from 27 to 32 weeks, and the birth weights ranged from 800 to 1220 g [Table 1]. Out of the initial 16 eyes, seven had stage 3 ROP, six had stage 3 plus disease (one of which was excluded due to retinal detachment), two had stage 4, and one had stage 4 with plus disease [Table 1]. Aggressive looking stage 3 without clear-cut signs of plus disease was included to improve the chances of success. Zone 2 was most commonly involved (12 eyes), with 1 eye involving zone 1, and 2 eyes involving zone 3 [Table 1]. Tortuousity and vessel dilatation were apparent before therapy. After injection, according to both consultants, reduced neovascular activity was observed in all 15 eyes and they remained stable during follow-up. No systemic side effects of bevacizumab were observed, and no further treatment was necessary.

## DISCUSSION

The reason for significant improvement but not complete resolution may involve the potency of VEGF in ROP. This mode of therapy seemed to give better results than similar cases in the past, treated with laser therapy alone. This is the first study on the efficacy of bevacizumab injections in infants with ROP in Pakistan. Recent studies have also shown promising results [Table 2]. The number of infants with ROP is increasing likely due to better medical management of premature infants worldwide. Another reason is that more infants are now eligible for ROP screening. Guidelines recently changed to recommend screening in premature infants 30 weeks or less post-menstrual age or compared to 28 weeks or less that was recommended previously.^11^ In developing countries however, fewer ophthalmologists are screening for ROP due to financial constraints. Given that poor outcomes, such as retinal detachment, are now less frequent with optimally timed laser treatment, there may be a presumption that a bad outcome was “caused” by deficient follow-up or ophthalmological care. Recent advances in photographic screening and digital transfer of images via email and file transfer protocol site on the world wide web, allows for second opinions, easier and quicker diagnosis, and patient awareness. Once ROP that requires treatment is diagnosed, the treatment should be performed within 3 days. In this study, the results indicated that neovascular activity decreased after bevacizumab injection in the entire cohort after initial laser therapy along with intravitreal bevacizumab injection. There was a in vascular dilatation and tortuosity decreased after bevacizumab injection in all eyes that had these conditions preoperatively. These observations strongly suggest that bevacizumab effectively reduces vascular activity in ROP. At our institution, ROP for the past 10 years (90–100 cases), was being treated solely with indirect argon laser.

**Table 2:** Summary of recent studies of the use of intravitreal bevacizumab with or without laser therapy for retinopathy of prematurity

Serial #	Author	Year of study	Number of children	Number of eyes	Remarks	Type of therapy
1	António Travassos	2007	3	6	In 24 h, all six injected eyes showed regression of the tunica vasculosa lentis and iris vessel engorgement and disappearance of iris rigidity. In addition, plus disease and retinal proliferation began to regress. None of the eyes required additional treatment.	Laser treatment followed by intravitreal bevacizumab
2	Chung EJ	2007	1	2	Treatment resulted in ROP regression, prompt resolution of plus signs, and neovascular proliferation in both of the two eyes.	Laser treatment followed by intravitreal bevacizumab
3	Mintz Hittner	2008	11	22	Intravitreal injection of bevacizumab was safe and effective in treating stage 3 ROP in zone I and posterior zone II in all 22 eyes.	Intravitreal bevacizumab as monotherapy
4	Shunji Kusaka	2008	14	23	Reduced neovascular activity was seen on fluorescein angiography in 14 eyes. In 3 eyes, tractional retinal detachment progressed after injection.	Laser treatment followed by intravitreal bevacizumab

According to our observations, from the current study there seems to be faster regression of the disease and a greater extent of regression and stabilization. Improvement has been observed in cases which when presented in the past and treated with indirect argon laser alone, did not show comparable regression of the disease.

Limitations of our study include the lack of preoperative and postoperative photographs to document the changes in the retina, due to lack of availability of a digital retinal introduced in Pakistan. The vascularity may have fluctuated with severity of plus disease and regressed automatically over a period of time. The retrospective nature of the study and the absence of a control group and a small number of patients present other limitations. However, due to due lack to data on this population (in Pakistan) we believe it is important to report the outcomes of our study. Bevacizumab reduced the vascular activity in severe ROP proving to be beneficial and we hope our results serve as the impetus for more research in this area.

Recent progress has been made beyond standard laser therapy, in the use of VEGF inhibitor bevacizumab, not only in choroidal neovascularization and age-related macular degeneration, but also in the treatment of ROP of stage 3 or higher. However, in comparing its effects in infants, it is important to keep in mind that we are dealing with structurally and physiologically immature retinas and little is known about the safety and possible adverse events associated with this drug in infants. Experimental data indicated that blocking all isoforms of VEGF can inhibit physiological neovascularization.^13^ Neonatal patients still need some vessel growth in other organs. However, treating patients with stage 3 ROP is significantly reduced the risk of retinal detachment. This strategy remains off-label and we recommend prospective, randomized, controlled clinical trials to compare the effect of laser treatment alone, of bevacizumab treatment alone, and of combined bevacizumab and laser treatment.
